# Different Neuroimaging Measurement Techniques for the Cerebellum in Alzheimer's Disease: VolBrain–Horos Comparison

**DOI:** 10.2174/0115734056394839250912054625

**Published:** 2025-09-19

**Authors:** Zumrut Dogan, Muhammed Emre Yuzer, Busra Zencirci, Fatih Uckardes, Erman Altunisik, Ali Haydar Baykan

**Affiliations:** 1 Department of Anatomy, Faculty of Medicine, Adiyaman University, Adiyaman, Turkey; 2 Department of Biostatistics and Medical Informatics, Faculty of Medicine, Adiyaman University, Adiyaman, Turkey; 3 Department of Neurology, Faculty of Medicine, Adiyaman University, Adiyaman, Turkey; 4 Department of Radiology, Faculty of Medicine, Adiyaman University, Adiyaman, Turkey

**Keywords:** Cerebellum, Horos, MRI, Neuroradiology, Bland‒altman plot, VolBrain, Alzheimer's disease, VolBrain–horos comparison

## Abstract

**Introduction:**

The use of magnetic resonance imaging (MRI), which has greater soft tissue contrast than other imaging modalities, has increased over the last 30 years. Studies have shown that MRI is frequently used for diagnosing neurodegenerative diseases. The incidence of Alzheimer's disease, a neurodegenerative condition, is increasing due to population aging and has a detrimental impact on quality of life. Volumetric changes in many important anatomical structures have been detected in magnetic resonance (MR) images of Alzheimer's disease patients. Various software programs, such as OsiriX, Horos, and VolBrain, are currently used to perform area and volume measurements in various brain structures. In this study, we compared the VolBrain and Horos applications for volume measurements of the cerebellum, whose relationship with Alzheimer's disease is not yet fully understood. We aimed to assess the consistency between the applications using various statistical methods and to highlight their respective advantages and disadvantages for researchers.

**Methods:**

This was a retrospective study. The patient group comprised 50 individuals with Alzheimer's disease aged 30–65 years. T1 MR images of 50 Alzheimer's disease patients were first acquired *via* the VolBrain program and then *via* the Horos program.

**Results:**

The applications used yielded almost identical measurement results, and no significant differences were observed.

**Discussion:**

Both applications have been found to produce consistent results. This indicates that the methods are reliable and that either application can be effectively used for the intended purpose.

**Conclusion:**

In conclusion, the choice between the two applications depends largely on the user’s data requirements, software preferences, and hardware capabilities. These factors play a decisive role in the selection process.

## 1 INTRODUCTION

Magnetic resonance imaging (MRI) produces cross-sectional images of the body *via* magnetism and radio waves [1]. Substantial developments have been made in this field over the past 40 years, and the use of MRI has been increasing in developed countries for the last 30 years [2]. Compared with other radiologic imaging modalities, MRI provides greater soft tissue contrast [3]. Recent studies have indicated that MRI is frequently used in the diagnosis of various neurodegenerative diseases [4-6]. Neurodegeneration is the progressive loss of structure and function in nerve cells and is a characteristic of several disorders, including Alzheimer's disease. MRI effec-tively reveals morphological differences and degeneration [7], which occur in various anatomical regions of the brain in neurodegenerative diseases [7-9]. However, whether neuro-degeneration occurs because of morphological changes or if morphometry changes because neurodegeneration occurs remains unclear. Clarifying this relationship will substantially advance the diagnosis and treatment protocols for neuro-degenerative diseases.

Alzheimer's disease is the most common neuro-degenerative disorder [10]. The globally aging population represents a significant risk factor for Alzheimer's disease. In addition to old age, some genetically inherited or envi-ronmental factors are also Alzheimer's disease risk factors, and the incidence of the disorder is continuously increasing worldwide. The pathological changes in the brain that define Alzheimer’s disease include extracellular amyloid-β plaques, intracellular neurofibrillary tangles, brain inflammation caused by synaptic and neuronal loss, and increased oxidative stress [11-14]. These neurodegenerative activities in the brain cause cell function loss and cell death, resulting in various cognitive and functional symptoms. Neurodegeneration is not static during the course of the disease; existing symptoms become more severe as Alzheimer’s disease progresses. In its advanced stages, Alzheimer's disease prevents patients from performing activities of daily living independently, and in the final stage, it leads to death [15-18]. Alzheimer's disease poses a serious threat to human health, and no accepted treatment methods exist. Current drug interventions alleviate Alzheimer’s disease symptoms but do not halt the neurodegeneration process [14, 19].

Alzheimer's disease can only be definitively diagnosed postmortem. Current Alzheimer's disease diagnostic protocols involve the sufficient follow-up of clinical symptoms, analysis of various biomarkers, and detection of disease-associated morphological distortions using brain MRI [20-22]. As reported in the literature, Alzheimer’s disease causes hippo-campal volume shrinkage, lobus temporalis and cerebral cortex atrophy, and sulcus cerebri and cerebral ventricle volume enlargement [14, 23-25]. The detection of these morpho-logical changes is important in the diagnosis of this disease. We hypothesize that the cerebellum, recognized as the brain’s coordination center and characterized by complex intracerebral communication pathways, may undergo morphometric changes with aging, potentially contributing to the development of neurodegenerative diseases such as Alzheimer's disease. Notably, volumetric calculations of this important anatomical structure will greatly contribute to the literature in this field. Thus, we compared different software programs for cerebellum volume measurements to determine their advantages and disadvantages, aiming to inform user preferences in research settings.

**Fig. (1):**
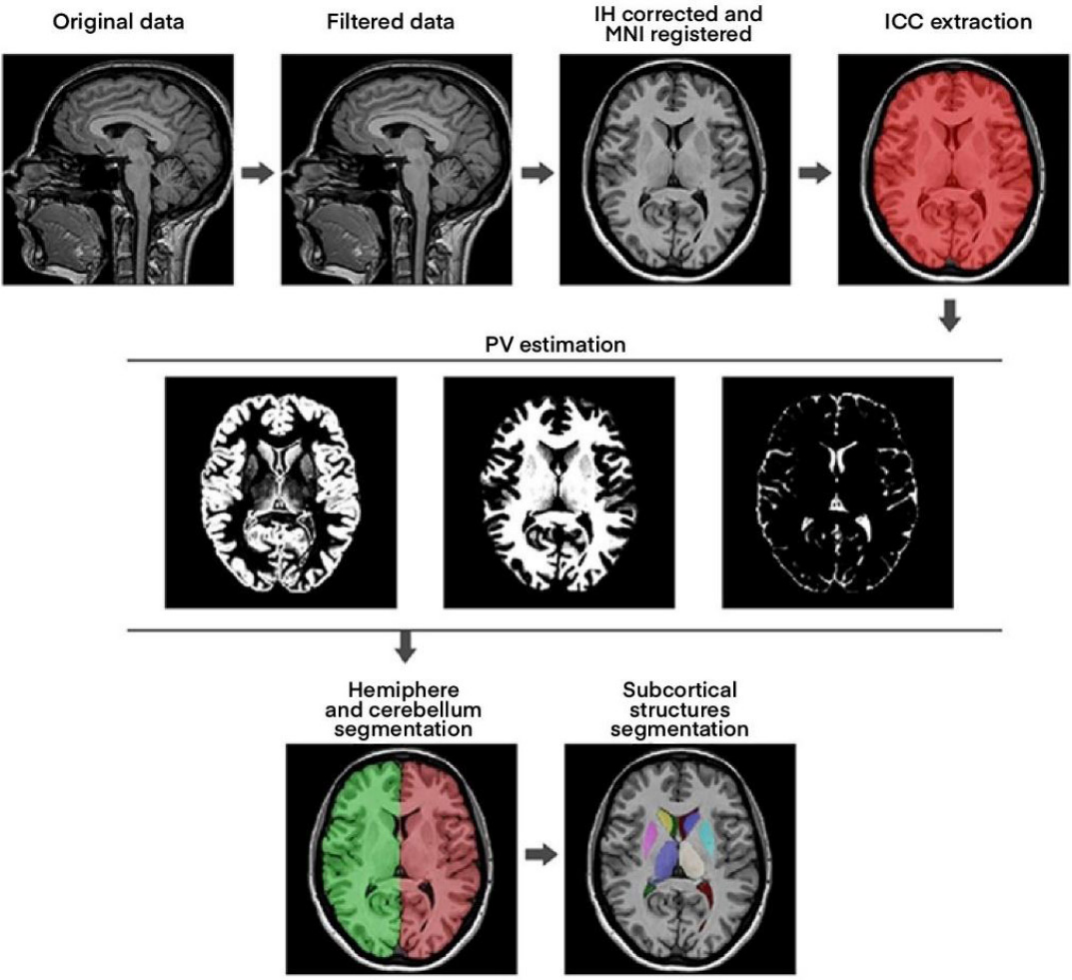
A schematic representation of the VolBrain image processing pipeline. The system performs automated MRI preprocessing, tissue segmentation, and quantitative analysis of brain structures, enabling standardized evaluation across datasets.

Numerous software tools are currently used to measure the area and volume of various brain structures. Area or volume losses can be detected using different methods. Measurement methods frequently reported in the literature include OsiriX, Horos, and VolBrain. VolBrain is an online software tool that allows researchers to conduct volume, segmentation, thickness, and asymmetry measurements of various brain areas from MR data without the need for any infrastructure at their local site. VolBrain provides users with a broad range of analytical capabilities through its specialized processing pipelines, including those for the brain, cerebellum, hippocampus, lesions, nuclei, and specific diseases (Fig. **1**) [26]. Horos version 1.1.7 (GNU L-GPL, Nimble Co LLC d/b/a Purview in Annapolis, MD, USA) is a free, open-source, Mac-based DICOM viewing and processing program with a user-friendly interface offering a wide range of image processing and evaluation methods. It is the open-source sibling of OsiriXTM and is a radiology-based morphometric viewer designed to facilitate the easy and intuitive visualization of CT and MR data. The Horos Project aims to develop a fully functional, 64-bit Mac OS X medical viewer for anatomical structures that can process DICOM-formatted images in 2D and 3D and perform spatial and volumetric measurements on the images [27].

The reliability of these two commonly used measurement methods, the accuracy of the data they produce, their relative ease of use, and the distinct features they offer researchers are important considerations. Researchers often compare data from two quantitative measurement methods to determine whether the methods can be used interchangeably. The Bland‒Altman (B&A) plot is the most common approach to such com-parisons. B&A plotting uses the standard deviation and mean of the differences between two measurement methods to demonstrate the consistencies and differences in the measure-ments. The x-axis shows the mean of the measurements, whereas the y-axis shows the difference between the two paired measurements. Thus, limits of agreement are established, and the consistency between these measurements is obtained [28, 29].

Degeneration of the hippocampus and cortex in Alzheimer's disease patients is unquestionably recognized in the literature. Cognitive and emotional problems in Alzheimer's patients are also well documented. Recent studies have shown that the cerebellum is important for cognitive and emotional functions. In cases of early-onset Alzheimer's disease (EOAD) [30-33], studies have indicated cerebellar involvement even at the early stages. Therefore, in this study, we measured the total cerebellar volume of Alzheimer's disease patients *via* the VolBrain and Horos methods. We assessed the anatomical structures susceptible to damage during neurodegeneration using the two different measurement methods to determine any significant differences between them and to elucidate their respective advantages and limitations.

## 2 METHODS

This was a retrospective study. The patient group comprised 50 individuals with Alzheimer's disease aged 30–65 years, 25 men and 25 women. All participants were diagnosed with EOAD by a neurologist at symptom onset and had mild (initial) disease. All cases were taken from the records of individuals who underwent brain MRI examinations between January 1, 2000, and January 1, 2022, at the Department of Radiology of Adıyaman University Training and Research Hospital. This study complied with SAGER (Sex and Gender Equity in Research) guidelines in order to consider the role of sex and gender in research design during participant selection, data collection, and analysis processes.

Patients with Parkinson's disease, spinocerebellar ataxia, Huntington's disease, vascular diseases, a history of stroke, brain tumors, and congenital and genetic disorders were excluded. Patients with a diagnosis of any neurodegenerative disease other than Alzheimer's disease were also excluded. Diseases that do not affect the brain or cerebellum globally, such as acute sinonasal disease and nonspecific headache, were not considered in the inclusion process.

Since this was a retrospective chart review, power analysis to determine the sample size was unnecessary, as concluded in consultation with a statistician. 3D T1 MR images with a 1 mm slice thickness (minimum of 30 samples per patient) were used. Images were not subjected to preprocessing before measure-ment with the Horos program. However, images filtered from the hospital system in DICOM format were converted to Nifti format before being uploaded into VolBrain as a software requirement. DICOM images have high spatial resolution before conversion and do not lose any resolution after conversion.

Volumetric measurements were performed on the MR images using Volbrain and Horos. T1 MR images of 50 Alzheimer's disease patients were first acquired *via* VolBrain and then *via* Horos. The B&A sample size method in Medcalc v20 was used to calculate the sample size of the study. The mean difference was 1, the standard deviation of the differences was 1, the maximum allowed difference between the methods was 4, the statistical power (1 - β) was 0.95, the two-way significance level was 0.05, and the sample size was n = 50.

### 2.1 Volbrain

**Fig. (2):**
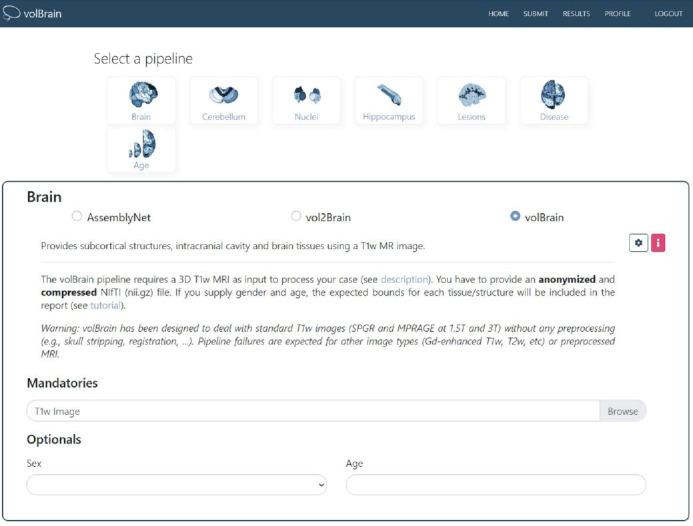
Conversion of DICOM images to NIfTI format and integration into the brain pipeline of VolBrain. The figure illustrates the workflow of transforming a DICOM medical image into NIfTI format, followed by its seamless integration into VolBrain's brain analysis pipeline for further processing, including brain segmentation, anatomical analysis, and statistical processing.

**Fig. (3):**
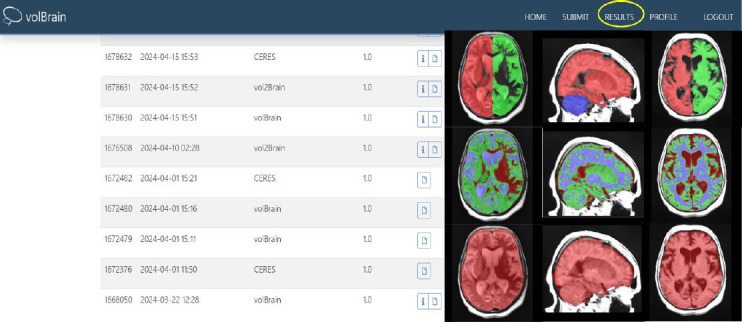
Data obtained from VolBrain for brain imaging analysis. The figure presents the results of the brain segmentation, anatomical analysis, and structural metrics obtained from the VolBrain platform. These outputs include volumetric measurements of different brain regions, 3D reconstructions, and statistical maps used for advanced neuroimaging analysis and interpretation.

For the VolBrain software, MR images in DICOM format were converted to Nifti format before being uploaded to the system. After conversion to Nifti format, since the total cerebellum volume was to be measured, the “brain pipeline” in VolBrain was selected, and the images were uploaded (Fig. **2**). Measurements were performed automatically by the software, and each image took approximately 5 minutes (Fig. **3**).

### 2.2 Horos

**Fig. (4):**
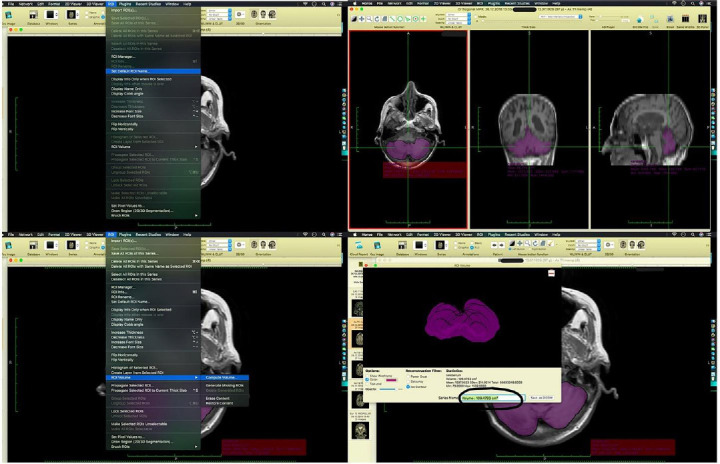
Implementation of ROI (region of interest) area selection, volumetric calculation, and 3D visualization of selected ROI areas in the Horos program. The figure illustrates the process of selecting and defining specific ROIs within brain imaging data *via* Horos. This includes manual and automated techniques for marking ROIs, followed by the extraction and analysis of quantitative metrics such as volume, intensity, and shape, which are essential for further neuroimaging analysis. The figure illustrates the process of performing volumetric measurements on defined ROIs and generating 3D schematizations to visualize their spatial and anatomical features. It highlights the steps involved in extracting quantitative data, such as volume and surface area from the segmented ROIs, followed by the creation of detailed 3D representations to enhance the understanding of structural characteristics within the brain or other relevant tissues.

The DICOM data of each patient were imported into Horos version 1.1.7 (GNU L-GPL, Nimble Co LLC d/b/a Purview in Annapolis, MD, USA). The ROI (cerebellum) in each slice of the black and white images was selected. The conformity of the selected areas to the anatomical position was examined across all three axes. Then, the program calculated the volume over each marked ROI (Fig. **4**). The same process was repeated for each patient.

### 2.3 Statistical Analyses

Statistical analyses were performed in MedCalc version 20.0 (2024 MedCalc Software Ltd.). The conformity of the data to a normal distribution was evaluated with the one-sample Kolmogorov‒Smirnov test. Independent two-sample *t* tests were used to compare the total cerebellum volume measurements obtained *via* the Volbrain and Horos programs. We also evaluated the comparison, agreement, reliability, and distribution of these two methods *via* various statistical methods. The methods used to assess differences; B&A plots, intraclass correlation coefficients, violin plots, and mountain plots. The significance level was set at *p* <0.05.

## 3 RESULTS

Volbrain and Horos were used for total cerebellum volume measurements in 50 individuals diagnosed with Alzheimer's disease. The details are presented in Table **1**.

As shown in Table **1**, the total cerebellar volume was 107.44 cm^3^ with VolBrain and 108.16 cm^3^ with Horos. The difference between the two measurement methods was less than 1 cm^3,^ and the standard deviations were almost identical. In the Kolmogorov–Smirnov normality test, *p* >0.05 indicated that the data were normally distributed. In addition, the independent two-sample *t* test (*p* >0.05) indicated that there was no significant difference between the methods.

In Table **2**, the letter a indicates the degree of consistency between the measurements, the letter b indicates the reliability of the single ratings, and the letter c indicates the reliability of the ratings' averages. The agreement between the results of the two methods was 98.59% (95% C1 = 97.53–99.20%). This finding indicates that both applications can be used inter-changeably.

**Fig. (5):**
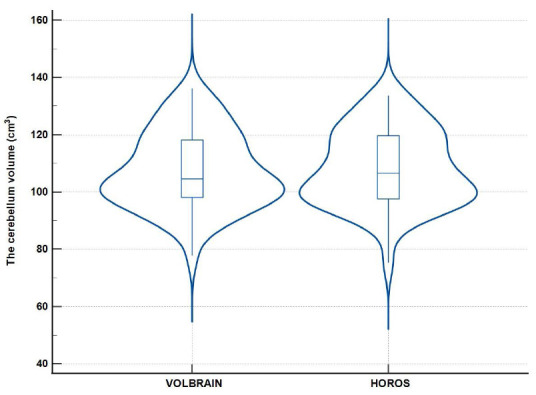
Data comparison *via* violin plot. The figure presents a violin plot comparing data distribution across different groups or conditions. It shows the density, median, and interquartile range of the data, providing a visual summary of the variations and central tendencies within each group. This visualization aids in identifying patterns, outliers, and differences between the groups for statistical analysis.

**Fig. (6):**
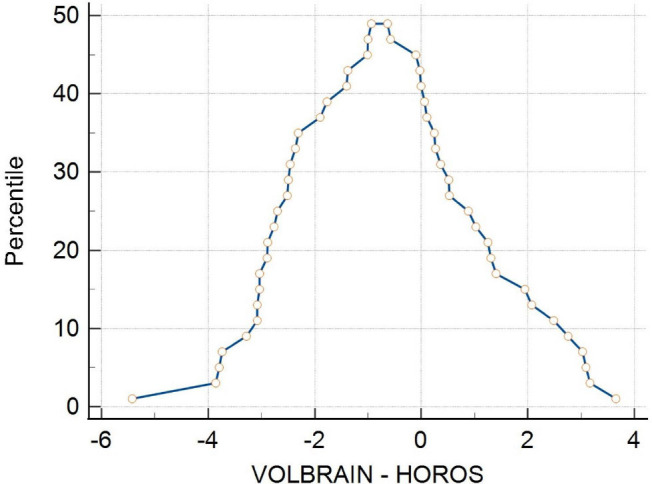
Data comparison *via* mountain plot. The figure illustrates the comparison of data distributions across different groups or conditions *via* a mountain plot. This visualization displays the density of data for each group, with the height of the 'mountains' representing the frequency of values. It provides a clear overview of the shape, spread, and central tendency of the data, facilitating the identification of differences, trends, and potential outliers between the groups.

Violin graphs were used to compare the distributions of the numerical values between the groups. The graphs obtained were very similar, indicating that the data from both treatments are consistent (Fig. **5**). The mountain plot illustrating the distribution of differences between the methods showed that the differences were centered near 0, indicating that the methods are compatible (Fig. **6**).

**Fig. (7):**
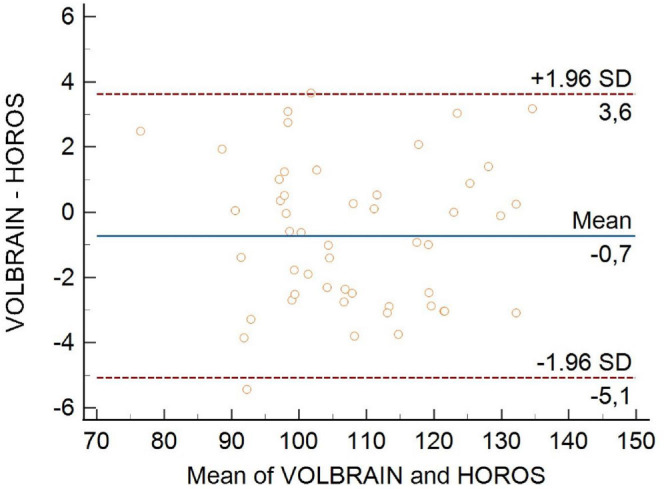
Data comparison *via* Bland‒Altman plot. The figure presents a Bland‒Altman plot comparing the two different measurement methods. This plot visualizes the agreement between the methods by plotting the differences against the averages of the two measurements. The figure highlights the limits of agreement, identifies potential biases, and assesses the precision and consistency between the two methods, providing insights into the reliability and concordance of the data.

**Fig. (8):**
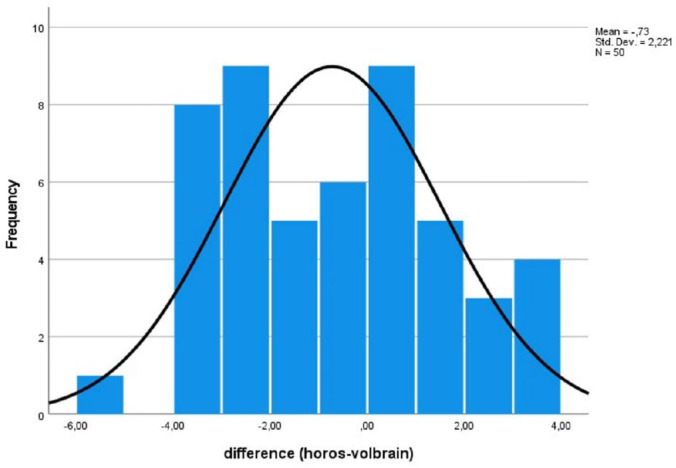
Difference descriptive statistics. The figure presents descriptive statistics comparing the differences between the two methods. It includes key metrics such as the mean, median, standard deviation, and range to summarize the distribution of differences. This analysis aids in understanding the central tendency, variability, and overall spread of the data, providing insights into the nature and extent of differences between the compared groups.

The B&A method was used to compare the two measurement methods, and we aimed to identify systematic differences or possible outliers (Fig. **7**). The B&A graph was used to calculate the 95% limits of agreement for each comparison, and the standard deviation (±1.96) was used to determine the difference in measurements between the two methods. Differences within ±1.96 standard deviations are not clinically significant; thus, the two methods can be used interchangeably. The B&A graph revealed a 0.7 cm^3^ difference between the two measurements (Fig. **7**). The x-axis shows the difference, whereas the y-axis shows the frequency. The greatest difference was 5.4 cm^3^. This measurement was 94.9 cm3 in Horos and 89.5 cm^3^ in VolBrain. Fourteen out of the 50 patients had a difference of less than 1 cm^3^ between their measurements. As shown in the B&A graph, the mean difference between the two measurement methods was 0.7 cm^3^ (Fig. **8**).

## 4 DISCUSSION

Alzheimer's disease, a neurodegenerative disease charac-terized by impaired quality of life, potential life-threatening complications, and limited treatment options, represents one of the major health challenges of our time. Diagnosis and treatment methods are diversifying with advances in technology. Recent studies have focused on the atrophy of anatomical formations in the brain in Alzheimer's disease. The basis of morphologic studies is radiology-based research [20, 22, 34, 35]. Anatomical imaging is important in the diagnosis of this disease. Therefore, the accuracy of the programs used in morphological evaluation is critical. A literature review revealed that there are no clear data on the advantages and disadvantages of these programs. Therefore, this study compared two different programs with different databases and file processing systems, providing a new perspective on early diagnosis methods. We hypothesize that conducting measurements on MR images of individuals with a family history of Alzheimer's disease or those in high-risk groups before the onset of clinical symptoms may become increasingly important for early diagnosis.

In recent years, the frequency of use of the VolBrain software has increased. Important data on this subject were obtained in the literature review. Walia *et al*. described 70 patients, 47 with younger-onset dementias and 23 with primary psychiatric disorders, 18 of whom had Alzheimer's disease. They analyzed the whole-brain volume and white and gray matter volumes of these patients *via* VolBrain software and reported that the disease caused atrophy in various morphometric areas of the brain [36]. Turamanlar *et al*. obtained cerebellar segmentations and lobular volumes of the cerebellum from 100 individuals aged 0–15 years *via* VolBrain software and presented similar data [37]. In addition to being used in the detection of damaged areas in neurodegenerative diseases such as Alzheimer's disease, MR images facilitate diagnosis through imaging and identifying deteriorated anatomical structures in many disease groups. Another VolBrain study automatically analyzed the total cerebellum volume in MR images of 26 adolescent idiopathic scoliosis patients using the software’s CERES pipeline [38]. These studies suggest that with the advancement of technology, the determination of deformation and atrophy in anatomical structures can be evaluated as an early diagnostic method in many disease groups, especially neurodegenerative diseases.

In this study, we determined that the average cerebellar volume of Alzheimer's patients was 107.44 cm^3^ *via* VolBrain software, whereas a similar study reported a volume of 118.40 cm^3^ [39]. In addition to determining the reliability of our measurements, we provided data that can be used to evaluate different MR-based measurement methods. No other study in which cerebellum images were measured *via* the Horos program was found in the literature. However, when similar studies were performed, Zamani *et al*. compared the volumes of cortical and subcortical brain segments *via* HIPS, VolBrain, CAT, and BrainSuite. Their study revealed a strong correlation between VolBrain and CAT, whereas no significant correlation was detected between these two methods and BrainSuite [40]. Koussis *et al*. analyzed various brain segments to compare NeuroQuant and VolBrain software and reported significant differences in all the brain segments tested except the hippocampus [41]. In a study of patients with glioma (the most common type of malignant brain tumor), Zeppa *et al*. analyzed pre- and postoperative tumor volumes. They used Horos for manual segmentation, SmartBrush (a tool in IPlan Cranial software (Brainlab, Feldkirchen, Germany)) for semiautomated segmentation, and BraTumIA software (NeuroImaging Tools and Resources Collaboratory) for automated segmentation. A comparison of volumes calculated with Horos and IPlan revealed strong agreement in both preoperative and postoperative images, whereas the agreement between BraTumIA and the other 2 techniques was strong in preoperative but not postoperative images [42].

VolBrain software enables faster and more user-friendly acquisition of measurements than Horos software. However, the images obtained *via* Volbrain are 2-D, whereas those obtained *via* Horos are both 2-D and 3-D. VolBrain software cannot measure MR images when the number of samples is insufficient, or the cross-sectional interval is high. In other words, the application's usability is weakened if appropriate MR images cannot be provided. Thus, VolBrain software cannot always be used. Horos, on the other hand, has no such restriction. The anatomy of the area to be measured must be well-known when using Horos because the user manually determines the measurement site at each slice or at a specified slice interval. Failure to correctly select the measurement site may reduce the reliability of the data obtained.

## CONCLUSION

This study revealed no significant differences between the measurement results obtained with VolBrain and Horos, indicating that both applications provide users with consistent data. The scope of our study was limited to cerebellum volume measurement, and there is no accepted standard cerebellum volume for Alzheimer's disease, limiting the software comparison. The finding that both software programs yield highly similar results serves as an important indicator of the reliability of other studies utilizing these tools. Our findings suggest that both software programs can be reliably employed in future research applications. The main purpose of this study was not to determine which software is superior but to demonstrate the reliability and repeatability of the measure-ment results. By identifying the advantages and disadvantages of both software programs, we aimed to make it easier for researchers to choose the appropriate software for future studies. Furthermore, we aimed to emphasize the importance of imaging methods in the early diagnosis of neurodegenerative disorders that impair the quality of life of both patients and their relatives, such as Alzheimer's disease.

Despite the valuable findings, this study has some limitations. The sample size was relatively small, which may limit the generalisability of the findings. In addition, cerebellar volume was the only parameter evaluated, and other critical brain regions associated with Alzheimer's disease were not included in the analysis. Furthermore, although both VolBrain and Horos software produced consistent results in this context, variability in measurement accuracy across different brain regions or patient populations may affect their overall applicability. Future studies with larger and more diverse cohorts and broader anatomical assessments are needed to further validate and extend these findings.

## Figures and Tables

**Table 1:** Descriptive statistics and normality test.

**Method**	**N**	**Arithmetic Mean**	**Standard Deviation**	**Min–Max**	**Kolmogorov–Smirnov**	**P^Ɵ^ value**
VolBrain	50	107.44 cm^3^	13.22 cm^3^	77.79 cm^3^– 136.14 cm^3^	D = 0.1065 *p* >0.10	0.7842
Horos	50	108.16 cm^3^	13.23 cm^3^	75.30 cm^3^– 133.70 cm^3^	D = 0.1160 *p* = 0.0899	

**Note:** P^Ɵ^: Independent two-sample *t* test.

**Table 2:** Interclass correlation coefficient.

-	**Intraclass Correlation^a^**	**95% Confidence Interval**
Single measures^b^	0.9859	0.9753 to 0.9920
Average measures^c^	0.9929	0.9875 to 0.9960

## Data Availability

The data and supportive information are available within the article.

## LIST OF ABBREVIATIONS

MRI: Magnetic Resonance Imaging
MR: Magnetic Resonance
B&A: The Bland‒Altman
EOAD: Early-Onset Alzheimer's Disease
SAGER: Sex and Gender Equity in Research
ROI: Region of Interest

## STANDARDS OF REPORTING

STROBE guidelines were followed.
